# Roles of ERK signaling pathway in regulating myelination of the peripheral nervous system

**DOI:** 10.3389/fnmol.2025.1617976

**Published:** 2025-06-13

**Authors:** Di Liu, Jingwei Zhou

**Affiliations:** Department of Endocrinology and Nephrology, Dongzhimen Hospital, The First Affiliated Hospital of Beijing University of Chinese Medicine, Beijing, China

**Keywords:** ERK, myelination, remyelination, Schwann cell, peripheral nervous system

## Abstract

Myelination of Schwann cells is a complex biological process that plays a crucial role in peripheral nervous system (PNS) development and repair. Recent studies have indicated that the extracellular signal-related kinase (ERK) signaling pathway participates in both developmental PNS myelination and remyelination. This review focuses on recent evidence identifying the roles of the ERK signaling pathway in regulating Schwann cell differentiation, myelination, and remyelination. In addition, the crosstalk between the ERK signaling pathway and other cellular signaling pathways that control Schwann cell myelination, such as c-Jun and Notch, are discussed. This review provides an overview of recent studies, revealing that dysregulated expression of the ERK signaling pathway participated in the pathogenesis of hereditary and acquired peripheral neuropathies.

## Introduction

To form the myelin sheath and insulation, which speeds up the transmission of electrical signals and offers metabolic and trophic support to nerve axons, Schwann cells spirally wrap around large-diameter axons at a 1:1 ratio in the peripheral nervous system (Feltri et al., 2016; Salzer, 2015). This fine-tuned process plays a central role in PNS development, as emphasized by inherited demyelinating neuropathies caused by malformation of the myelin sheath. Moreover, regeneration following the peripheral nerve injury also requires remyelination of the regenerated axons, which is critical for the successful functional restoration of PNS. Functionally impaired intact axons with abnormal myelination are a key mechanism in many human peripheral neuropathies (Höke, 2006). Therefore, understanding the underlying mechanisms that regulate myelination holds significance in elucidating the pathogenesis of peripheral neuropathies and developing new therapeutic strategies.

Recent studies have reported that the extracellular signal-related kinase (ERK) signaling pathway actively participates in developmental PNS myelination and peripheral nerve regeneration (Napoli et al., 2012; Sheean et al., 2014). Therefore, this review gives attention to the roles of the ERK signaling pathway in regulating PNS myelin development as well as the remyelination process after damage. The crosstalk between the ERK signaling pathway and other cellular signaling pathways that control Schwann cell myelination is discussed. Finally, the roles of the ERK signaling pathway in developing both inherited and acquired peripheral neuropathies are reviewed.

## The ERK1/2 signaling pathway

ERK1/2, as constituents of the mitogen-activated protein kinase (MAPK) family, are extensively expressed hydrophilic non-receptor proteins that contribute to the Ras–Raf–MEK–ERK signaling pathway (Roskoski, 2012). Ras-GTP initiates the activation of Raf kinases, which then catalyze MEK1 and MEK2 phosphorylation and stimulation (Raman et al., 2007). MEK1/2 are dual-specificity protein kinases that promote tyrosine and threonine phosphorylation in ERK1 and ERK2, their sole recognized physiological substrates (Raman et al., 2007). The ERK signaling pathway has long been acknowledged as a regulator of numerous processes, such as cell survival, differentiation, proliferation, adhesion, cycle progression, and metabolism (Wortzel and Seger, 2011; Cargnello and Roux, 2011; Lefloch et al., 2009; Gonsalvez et al., 2016). The disruption of this pathway can potentially result in a range of diseases, such as neurodegenerative disorders, metabolic disease, cancer, cardiac disorders, and inflammatory disease (Pylayeva-Gupta et al., 2011; Subramaniam and Unsicker, 2010; Steelman et al., 2011; Kim and Choi, 2010).

Previous investigations showed that various cellular stressors, such as cytokines, bradykinin, insulin, epidermal growth factor (EGF), fibroblast growth factor, insulin-like growth factor-1, platelet-derived growth factor, and others, can stimulate ERK signaling (Raman et al., 2007). Furthermore, for myelination, the ERK signaling pathway participated in controlling the promyelinating effects of numerous growth factors, such as brain-derived neurotrophic factor (BDNF), fibroblast growth factor, and EGF family proteins like neuregulin 1 (NRG-1) type III (Gonsalvez et al., 2016; Newbern and Birchmeier, 2010; Xiao et al., 2012; Cui and Almazan, 2007; Syed et al., 2010).

## ERK signaling pathway in Schwann cell differentiation

In the initial stages of embryonic development, a small, temporary group of cells known as neural crest cells, isolated from the neural tube at the time of neural tube closure, have been identified to originate Schwann cell precursors (SCPs) (Scherer, 1997). At the initial transitional stage in the Schwann cell lineage, SCPs serve as the glial cells of early embryonic neurons and provide the immature Schwann cells (Monk et al., 2015). Schwann cells, which are present in mature nerve trunks, undergo differentiation into myelinating and non-myelinating varieties during birth (Salzer, 2015). The earliest commitment toward myelin synthesis is expected to occur during the shift from immature Schwann cells to promyelinating Schwann cells, which is why this phase is crucial. Schwann cell differentiation and maturation are precisely controlled, relying on the synchronized expression of differentiation-enhancing genes and inhibition of negative regulators (Monk et al., 2015).

**Figure 1:**
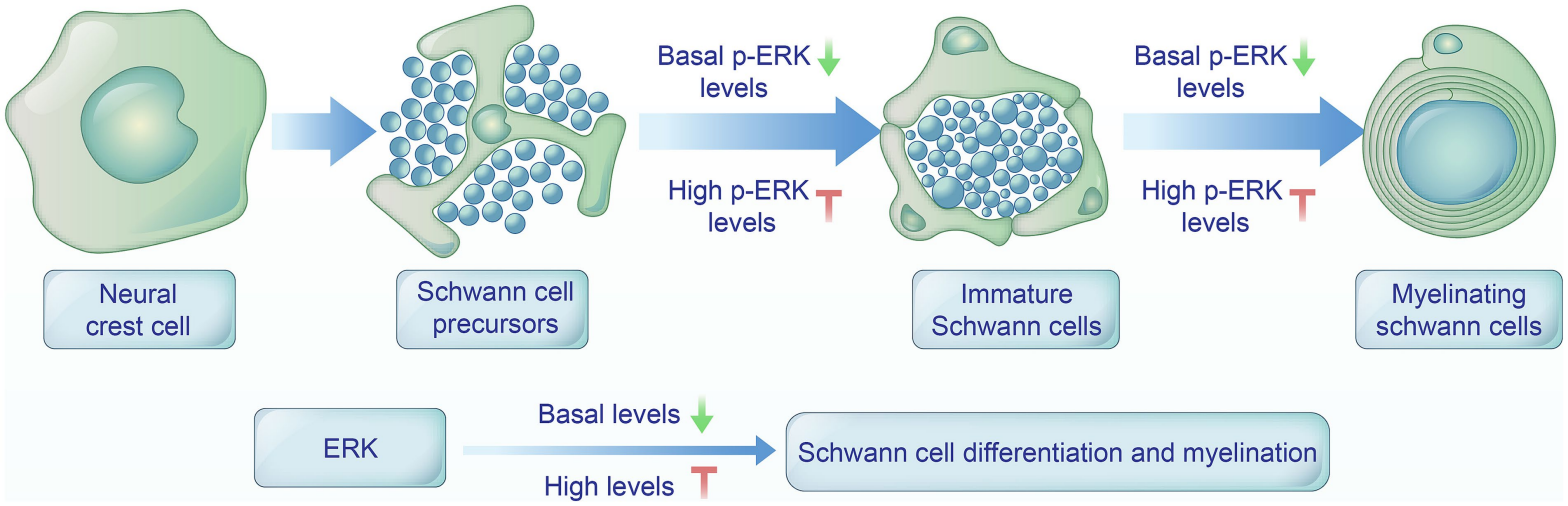
Roles of ERK signaling pathway in regulating Schwann cell differentiation and myelination. Schwann cell precursors (SCPs) are the first transitional stage in the Schwann cell lineage derived from neural crest cells. SCPs cease migration and develop into immature Schwann cells. Immature Schwann cells envelop single large-diameter axons and differentiate into myelinating Schwann cells. During the developmental process, basal levels of ERK pathway activity are indispensable for the differentiation of precursors and myelin maintenance, while high levels of ERK activation negatively regulate Schwann cell differentiation and myelination.

One of the most important regulators of Schwann cell differentiation and proliferation is ERK signaling. In primary rat Schwann cells, strong activation of ERK was found to suppress promyelinating markers of Schwann cells, while this suppressive effect was entirely abolished by blocking ERK signaling, suggesting that the ERK pathway suppresses Schwann cell differentiation (Harrisingh et al., 2004). Concurrently, another study also revealed that ERK pathway activation can block Schwann cell differentiation and actively drive the dedifferentiation process (Ogata et al., 2004). Moreover, in mouse primary Schwann cells, the ERK1/2 phosphorylation was found to be linked to elevated mRNA levels of markers for undifferentiated Schwann cells such as Krox-24 and Scip (Nadra et al., 2008). A previous study reported that the suppression effect of adenosine on PDGF-induced Schwann cell proliferation was mediated through the stimulation of the ERK signaling pathway (Stevens et al., 2004). Collectively, these outcomes provide convincing evidence that ERK signaling exerts negative effects on Schwann cell differentiation and proliferation (Figure 1).

However, recent research has shown that the ERK pathway is essential for Schwann cell differentiation during development (Figure 1). A significant reduction of SCPs was detected in the peripheral nerve of ERK1 null allele embryos, suggesting that ERK1/2 is critical for SCP development of the peripheral nerve *in vivo* (Newbern et al., 2011). The neuregulin/ErbB axis has an essential function in Schwann cell differentiation (Newbern and Birchmeier, 2010). This study also stated that ERK1/2 is essential for mediating the impacts of NRG-1 on Schwann cells *in vivo*. Furthermore, conditional deletion of Shp2, an upstream ERK/MAPK stimulator, in neural crest cells leads to defects in Schwann cell development, including cell proliferation, migration, and differentiation (Grossmann et al., 2009). Nevertheless, the results from a recent study indicated that small molecule inhibitors targeting MEK and ERK did not influence the expression levels of Oct6 and EGR2, which are essential transcription factors of Schwann cell differentiation (Park et al., 2021). Nonetheless, the roles of the ERK pathway on Schwann cell differentiation remain incompletely understood.

Different studies have concluded that ERK signaling has both positive and negative effects on Schwann cell differentiation. The reconciling explanation could be that distinct levels or duration of ERK activity would define the state of Schwann cell differentiation. Low or basal activity of ERK would be required for Schwann cell differentiation while high ERK levels would drive dedifferentiation and proliferation (Newbern and Snider, 2012). ERK signaling seems to play a pro-differentiating role during development and a pro-dedifferentiating role after nerve injury. This dualistic role may depend on the lasting of activation. Following peripheral nerve injury, the levels of phosphorylated ERK/MAPK in the distal nerve rise more than threefold and remain elevated in the Büngner bands for up to a month (Sheu et al., 2000). Transient activation of ERK signaling during developmental stages may be linked to positive differentiation modulation, while long-term activation ERK signaling following nerve injury may lead to its negative modulation (Castelnovo et al., 2017). Another possibility is that ERK signaling pathway may interact with other pathways that regulate Schwann cell differentiation. The development of Schwann cells follows a structured sequence from the neural crest to fully differentiated Schwann cells, involving integration of both cell autonomous and non-cell autonomous signaling mechanisms. For example, during nerve development, cAMP/PKA signaling directs ERK signaling to facilitate Schwann cell differentiation, while in damaged adult nerves, lower cAMP levels link ERK signaling to Schwann cell dedifferentiation (Arthur-Farraj et al., 2011). Nonetheless, the roles of the ERK pathway on Schwann cell differentiation remain incompletely understood.

## ERK signaling pathway in myelination by Schwann cells

**Figure 2:**
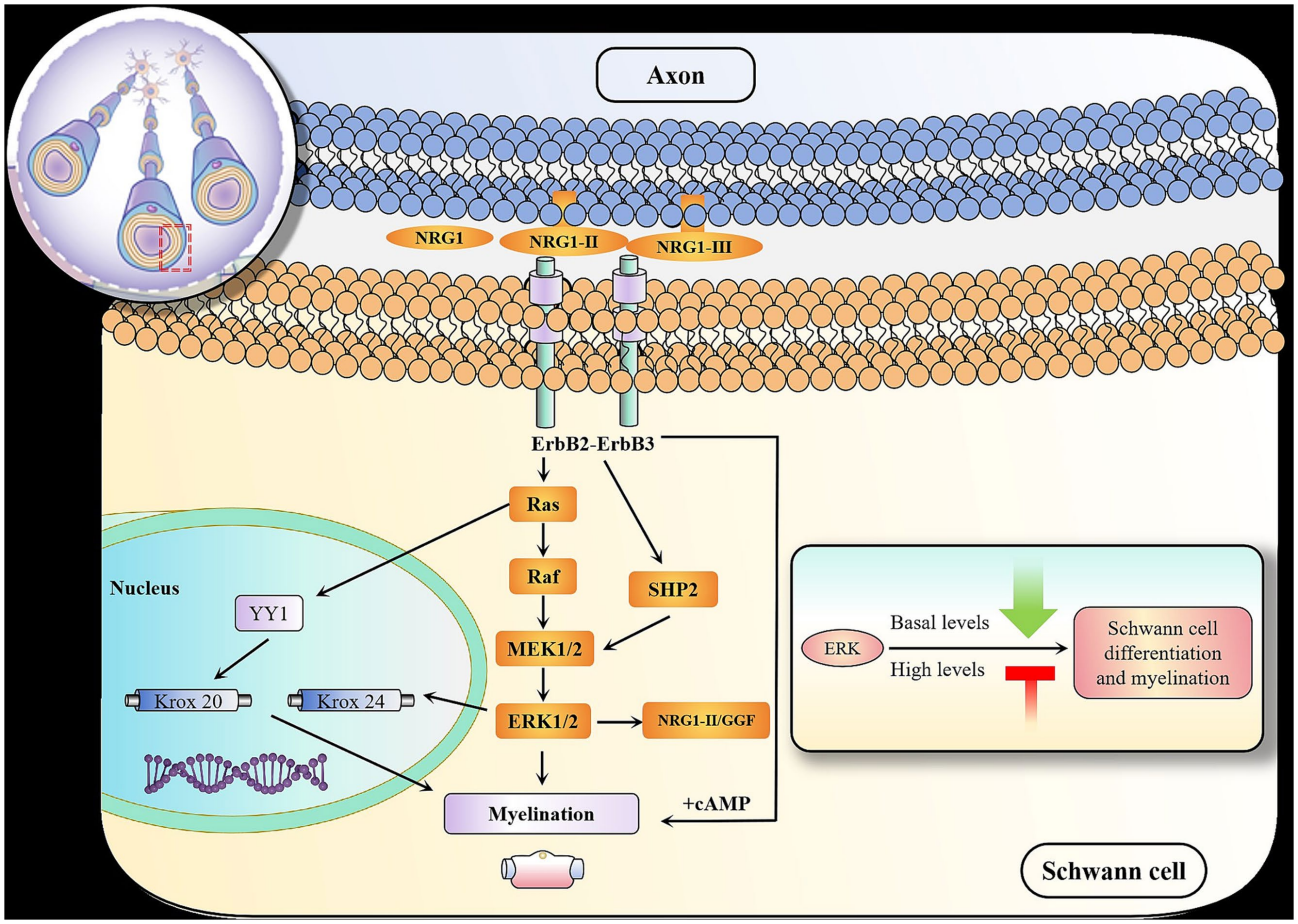
Schematic of ERK signaling pathway that regulate Schwann cell myelination. Schwann cell myelination is a process strongly dependent on instructive signals provided by the axons. The major axonal signals include type III NRG1, which signal via erbB receptors. Following NGF binding to tropomyosin, receptor kinase activates Ras. The MEK1/2-dependent phosphorylation of ERK1/2 induces their nuclear translocation, enabling ERK1/2 to potentially phosphorylate and/or stabilize transcription factors and proteins that then alter gene expression. MEK, the kinase directly upstream of ERK1/2 induce the phosphorylation of YY1. YY1 drive peripheral myelination transcription, such as Krox20. ERK1/2 phosphorylation can upregulate Krox-24 expression. The phosphatase Shp2, which activates the ERK1/2 signaling pathway, is necessary for Schwann cell differentiation and myelination. In addition, MEK/ERK can collaborate with soluble Nrg1 type II (GGF) to offer extra inhibitory signals for myelination. During the myelination process, basal levels of ERK activity are necessary for differentiation of precursors while high levels of ERK activity negatively regulate Schwann cell differentiation and myelination.

Schwann cell myelination involves the recognizing of crucial external signals, the activation of intracellular pathways, and the transcriptional and epigenetic programs that synergistically promote the myelinating phenotype (Salzer, 2015). Growing evidence supports the role of the ERK signaling pathway in Schwann cell myelination (Figures 1, 2). Activation of the ERK signaling pathway was observed to downregulate myelin proteins in Schwann cell cultures expressing myelin proteins induced by cAMP, as well as cause demyelination in DRG neuron/Schwann cell co-cultures (Harrisingh et al., 2004). Another study investigating neuron/Schwann cell co-cultures also indicated that the endogenous Mek/ERK action in co-cultures is likely to collaborate with soluble Nrg1 type II (GGF) to offer extra inhibitory signals for myelination. GGF inhibitory effect on myelination is linked to the activation of c-Jun induced by the MEK/ERK signaling pathway (Syed et al., 2010). This study also demonstrated that a high concentration of soluble Nrg1 type III suppresses myelination depending on MEK/ERK. Furthermore, *in vivo* evidence indicates that a significant rise in the MEK/ERK/c-Jun pathway is associated with a decrease in the myelin gene expression as well as a delay in Schwann cell differentiation (Scapin et al., 2020). Collectively, these researches indicate that high levels of phosphorylation of the ERK signaling pathway negatively regulate Schwann cell myelination.

Conversely, ERK signaling is required for PNS myelination. Newbern et al. (2011) reported that the absence of ERK 1/2 in SCPs resulted in differentiation disruption and significant hypomyelination of axons, highlighting the importance of ERK 1/2 for the progression of the myelinating Schwann cell lineage following initial specification. Moreover, the MEK-dependent transcription factor Yy has been proven to be critical in the process of peripheral myelination (He et al., 2010). In addition, conditional mutation of Shp2 in myelinating Schwann cells can cause hypomyelination and is found to be linked to decreased ERK1/2 phosphorylation (Grossmann et al., 2009). Finally, recent studies indicate that sustained stimulation of the ERK signaling pathway can override the signals that terminate myelination, leading to continuous myelin growth (Sheean et al., 2014; Ishii et al., 2013). It has been observed that the stimulation of ERK signaling can compensate for the lack of ErbB3/Shp2 signaling pathway during Schwann cell growth and myelination (Sheean et al., 2014). These outcomes indicate that the ERK signaling pathway is a conserved mechanism that promotes PNS developmental myelination (Figures 1, 2).

At first, different levels or duration of ERK activity may elicit distinct responses, potentially explaining these seemingly paradoxical observations. The basal levels of ERK pathway is necessary for the differentiation of precursors and myelin maintenance, whereas high levels of ERK activation negatively regulate Schwann cell differentiation and myelination (Newbern and Snider, 2012). Therefore, the state of Schwann cell development might be defined by levels of ERK activity. Secondly, there are differences in the regulation of developmental myelination and remyelination. The ERK signaling is activated by NRG-1 and promotes myelination during development, but sustain activation of ERK by NRG-1 inhibits myelination (Scherer, 1997). It is also possible that the impact of the ERK signaling pathway on neural repair is related to the intensity and duration of its expression. In the early stages of nerve injury, the ERKsignaling can promote myelin clearance. However, during the advanced stages of nerve repair, the ERK signaling can inhibit myelination, which hinders the remyelination of regenerating axons and delays the restoration of neural function (Cervellini et al., 2018). Thirdly, it has also been suggested that other signaling pathways may interact with ERK signaling to influence the myelination by Schwann cells. For example, during active myelination, ERK signaling is dependent on mTOR signaling to drive the growth of the myelin sheath and regulate myelin thickness (Ishii et al., 2021). Furthermore, the transition into the myelinating Schwann cell stage requires accurate asymmetrical Schwann cell polarization created upon axo-glial contact. Axon-glial communications play a vital role in regulating the myelination process and axonal cytoskeleton by altering important nodes within intracellular signaling pathways and the transcriptional network of neuron–glia (Rao and Pearse, 2016). Axon derived NRG1-type III can bind the ErbB2/3 receptor on Schwann cells and supresses Nrg1-type I expression by MEK/ERK signaling. In the context of Charcot–Marie-Tooth (CMT) disease, the disrupted axon-glia communication reduces PI3K/AKT signaling activity, which in turn causes MEK/ERK hyperactivation. The imbalanced activity of PI3K/AKT and MEK/ERK signaling pathways triggers the transcription of Schwann cell immature and repair markers, resulting in impaired Schwann cell differentiation and myelination. When the activity of PI3K/AKT and MEK/ERK signaling pathways is balanced, thereby maintaining Schwann cells in their differentiation state. In addition, the differentiation of Schwann cells is also influenced by signals coming from the extracellular matrix (ECM) externally. ECM-derived collagen VI negatively ERK activation and inhibits myelination (Chen et al., 2013). However, clarifying the roles of the ERK signaling pathway and determining how ERK signaling operate with other signaling pathways control changes in the transcriptional network that regulates Schwann cell behavior will be challenging.

## ERK signaling pathway in remyelination by Schwann cells

Following axotomy or nerve crush injury, the distal stump of the peripheral nerve undergoes Wallerian degeneration. Wallerian degeneration involves several phases of structural changes: alterations of initial axons, recruitment of macrophages, removal of myelin and axon debris, responses of Schwann cells, and actions of the other neural cells (Chen et al., 2007). When a nerve is transected, the axons at the distal stump experience granular degeneration, which is then followed by the recruitment of circulating macrophages into the endoneurium to phagocytize debris of myelin and axons. At injury sites, Schwann cells dedifferentiate and proliferate, forming Büngner bands to facilitate a supportive environment for axon regeneration and promote remyelination (Jessen et al., 2015). The growth of regenerating axons occurs along Büngner bands, leading to an increase in their diameter. The process of remyelination starts once Schwann cells encounter the regrowing axons. Hereditary and acquired peripheral neuropathies are characterized by demyelination accompanied by incomplete remyelination (Höke, 2006).

**Figure 3:**
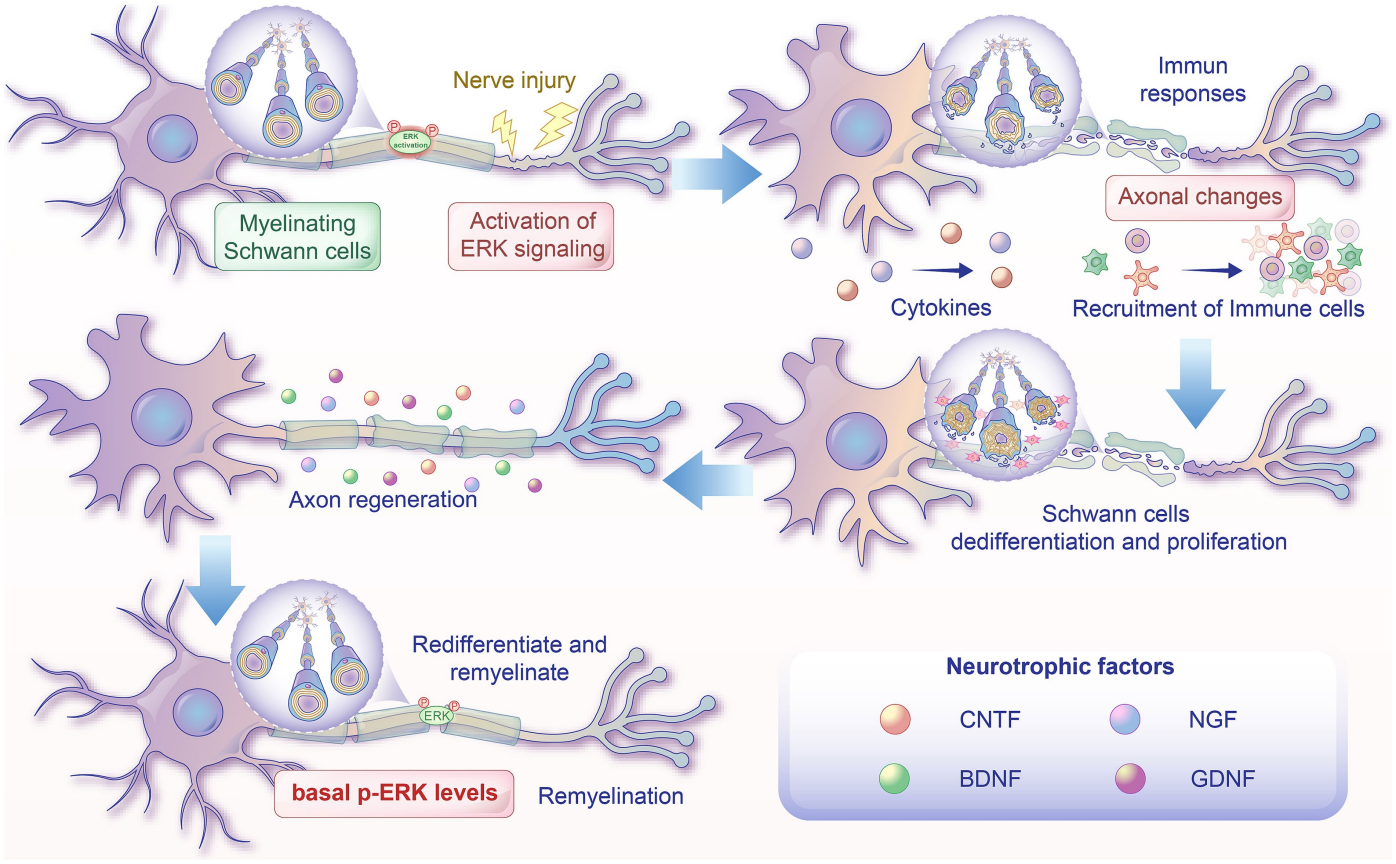
Roles of the ERK signaling pathway in regulating remyelination. After nerve crush injury or axotomy, the distal stump of the peripheral nerve undergoes Wallerian degeneration, which includes early degenerative axonal changes, immune cell recruitment, Schwann cell dedifferentiation and proliferation, and axon regeneration and remyelination. Nerve injury results in a rapid activation of ERK signaling in Schwann cells. A number of cytokines are upregulated, and a variety of immune cells are recruited following the activation of the ERK signaling pathway. ERK activation results in Schwann cell dedifferentiation and proliferation. The ERK signaling pathway also participates in regulating neurotrophic factor expressions and axonal outgrowth. After phosphorylated ERK levels return to basal levels, dedifferentiated Schwann cells redifferentiate and remyelinate.

*In vivo* and *in vitro* experiments reveal that the ERK signaling pathway has a central function in peripheral nerve regeneration, including regulating immune responses, Schwann cell dedifferentiation and proliferation, neurotrophic factors, and axon regeneration and remyelination (Figure 3).

### Immune responses

The recruitment of immune cells plays an essential role in clearing axon and myelin debris and supporting subsequent revascularization of damaged nerves. The ERK signaling pathway has been found to participate in regulating inflammatory responses after nerve injury. *In vivo*, following the stimulation of the ERK signaling pathway, there was a significant rise in the count of macrophages, neutrophils, mast cells and T cells in the nerve, which are all recruited after nerve injury (Napoli et al., 2012). Furthermore, in injured nerves, the process of immune cell recruitment can be blocked by highly selective ERK signaling inhibitors. Similarly, conditioned media from Schwann cells treated with an ERK signaling activator also effectively recruited immune cells. A number of cytokines, including c-kit ligand, MCP-1, IL11, and Cxcl10, were upregulated after stimulation of ERK signaling in dedifferentiated Schwann cells. In addition, in the nerves of a mouse model for Charcot–Marie Tooth neuropathy, the activation of ERK1/2 was found to be directly associated with the elevated secretion of the macrophage-attachment cytokine MCP-1 (Fischer et al., 2008). Clearance of myelin debris following acute demyelination is crucial for functional recovery after nerve injury. Functioning as “amateur” phagocytes, microvascular endothelial cells have been proven to engulf and clear myelin debris, which enhances inflammatory response, vigorous angiogenesis, and ongoing fibrosis. Furthermore, ERK signaling is also involved in regulating myelin debris that triggers endothelial-to-mesenchymal transition in a dose-dependent way and supports the migration of endothelial cells (Yao et al., 2023).

### Schwann cells dedifferentiation and proliferation

Schwann cell dedifferentiation and proliferation are significantly associated with successful nerve regeneration (Jessen et al., 2015). In addition, it was observed *in vivo* axotomy and *in vitro* culture of nerve segments that there was a rapid rise in ERK1/2 phosphorylation in Schwann cells, which subsequently caused a corresponding elevation in Schwann cell proliferation at ERK1/2 stimulation sites, which can be inhibited by ERK1/2 inhibitor (Martensson et al., 2007). Likewise, active ERK was mainly located in Schwann cells that form Büngner bands in the distal sciatic nerve segments (Agthong et al., 2006). Following a crush injury to the sciatic nerve, Schwann cells exhibit increased expressions of Epo and EpoR (Li et al., 2005). The application of exogenous Epo to damaged sciatic nerves or primary cultures of Schwann cells promotes Schwann cell proliferation by activating ERK/MAPK. Conversely, metalloproteinase-9, production of which was increased markedly by Schwann cells after nerve damage, was demonstrated to suppress Schwann cell proliferation and mitogenic activity through selective activation of ERK1/2 (Chattopadhyay and Shubayev, 2009). Furthermore, following the transfection of DRG neuron/Schwann cell co-cultures, active ERK1/2 led to Schwann cell dedifferentiation (Harrisingh et al., 2004). In a recent *in vivo* study, it was found that 3 days of increased ERK/MAPK phosphorylation notably induced Schwann cell dedifferentiation, and proliferating Schwann cell progenitors in the nerve was markedly increased (Napoli et al., 2012). This study provides convincing evidence that the RAF/MEK/ERK pathway activation alone can successfully induce Schwann cell dedifferentiation *in vivo*, even in nerves lacking damaged axons. Concurrently, a study reported that rapamycin repaired damaged nerve cells and neurological function by controlling Schwann cell proliferation via the ERK signaling pathway (Liu et al., 2020). Following peripheral nerve damage, the protein kinase C *α* (PKCα) expression, a serine/threonine kinase in SCs, was markedly increased. *In vivo* and *in vitro* culture trials further illustrated that PKCα significantly promoted Schwann cell migration by inducing the stimulation of the ERK signaling pathway. These outcomes suggest a potential mechanism for ERK to control Schwann cell proliferation after peripheral nerve injury (Li et al., 2020).

### Neurotrophic factors

Following peripheral nerve damage, the release of neurotrophic factors is crucial for neuronal survival and efficient nerve regeneration (Richner et al., 2014). Nerve damage triggers the neurotrophic factors’ expression, including NGF, GDNF, and BDNF. The sequential and overlapping induction of NGF, GDNF, and BDNF expression in the distal stump correlates with the sustained activation of ERK, indicating the essential role of ERK activation in establishing an extracellular environment that facilitates regeneration at the distal end of transected nerves (Abe et al., 2001). In contrast, markedly lower levels of CNTF expression were found in nerves that were injured and regenerating (Friedman et al., 1992). Experiments further revealed that blocking the ERK signaling pathway caused a significant elevation in CNTF expression in cultured IMS32 cells; moreover, there was an inverse association between CNTF expression and ERK activity in the Schwann cells of the sciatic nerve (Abe et al., 2001). As a part of the neurotrophin family, neurotrophin-3 (NT-3) is crucial for the development, upkeep, and survival of neurons within the nervous system. A recent study reported that NT-3 supported the regeneration of peripheral nerves by sustaining Schwann cells in a repair state after prolonged denervation through the TrkC/ERK/c-Jun signaling pathway (Xu et al., 2023). Collectively, these outcomes indicate that the ERK signaling pathway participates in regulating neurotrophic factors during peripheral nerve repair.

### Axon regeneration

Axon regeneration is also a key process for effective nerve regeneration. Increasing data indicates that the ERK signaling pathway has a function in controlling axon degeneration, sprouting, and regrowth after nerve injury. Research indicates that the suppression of the ERK pathway through the proteasome is involved in Wallerian degeneration of injured axons and axonal pruning in the absence of local NGF (Macinnis and Campenot, 2005). ERK signaling pathway not only promotes axon growth but also triggers the anti-apoptotic protein Bcl-2 through CREB to support axonal survival (Akram et al., 2022). Moreover, an *in vitro* study has revealed that PD98059, an inhibitor of MEK1/ERK1/2, almost fully inhibited neuron sprouting in damaged PC12 cells, indicating that MEK1/ERK1/2 has a crucial function in neurite re-formation (Waetzig and Herdegen, 2005). After peripheral nerve injury, the inhibitory microenvironment associated with myelin restricts nerve regeneration. A recent study revealed that ERK signaling is required for the regulation of axonal microenvironment. Blocking the EGFR-ERK pathway was shown to enhance TRIM32 expression, which can counteract the inhibitory effects of the microenvironment on the neuronal differentiation of neural stem cells and support neurogenesis (Ma et al., 2017). Following peripheral nerve damage, administering EGFR-ERK inhibitors enhances the neurogenesis in nestin(+) cells activated by the injury and improves functional recovery (Xue et al., 2020). Consistent with the pivotal role of ERK signaling in axon regeneration, a study from Tsuda revealed lower levels of p-ERK 1/2 in unrepaired transected nerves compared to the immediate or delayed repair groups (Tsuda et al., 2011). Notably, the study also proved that there was a correlation between axonal outgrowth length and p-ERK 1/2 expression at the lesion site and in the distal nerve segment. Moreover, the outcomes from in vitro and *in vivo* trials validated that the ERK pathway could promote myelinated axonal regrowth (Yang et al., 2023). A recent study from Tsuda showed that In chicken DRG, the non-integrin laminin receptor (LamR) promoted axonal outgrowth by regulating the Akt and ERK pathways, both of which are important for controlling axonal outgrowth (Mrówczyńska et al., 2024). These studies suggest that ERK plays an essential role in axon regeneration after nerve damage.

### Peripheral nerve remyelination

Functional peripheral nerve regeneration necessitates remyelination of the regenerated axons by Schwann cells. Growing evidence indicates that the ERK signaling pathway plays negative regulatory roles in remyelination. A study using DRG neuron/Schwann cell co-cultures indicated that ERK signaling pathway activation resulted in a marked decrease in mRNA levels of Protein zero (P0), peripheral myelin protein-22 (PMP22), and myelin basic protein (MBP), along with the myelin sheath breakdown (Harrisingh et al., 2004). Consistent with this report, a study employing an innovative transgenic mouse model that facilitates Schwann cell-specific, reversible ERK/MAPK stimulation also indicated that ERK signaling activation resulted in a rapid reduction in expressing myelin genes, P0, MBP, and periaxin, causing substantial nerve demyelination and motor/proprioceptive impairments during behavioral testing (Napoli et al., 2012). The observation of remyelination and motor recovery following the normalization of ERK signaling activity implies that a subsequent decrease in ERK/MAPK activity may be essential for remyelination (Napoli et al., 2012). Moreover, an *in vivo* study revealed that autocrine or paracrine NRG1 type I signaling by Schwann cells promotes efficient remyelination after damage, whereas axonal NRG1 type III signaling to myelinating Schwann cells continuously suppresses Nrg1 type I transcription via the ERK1/2 signaling pathway (Stassart et al., 2013).

## Crosstalk between ERK signaling pathway and other myelination regulators

**Figure 4:**
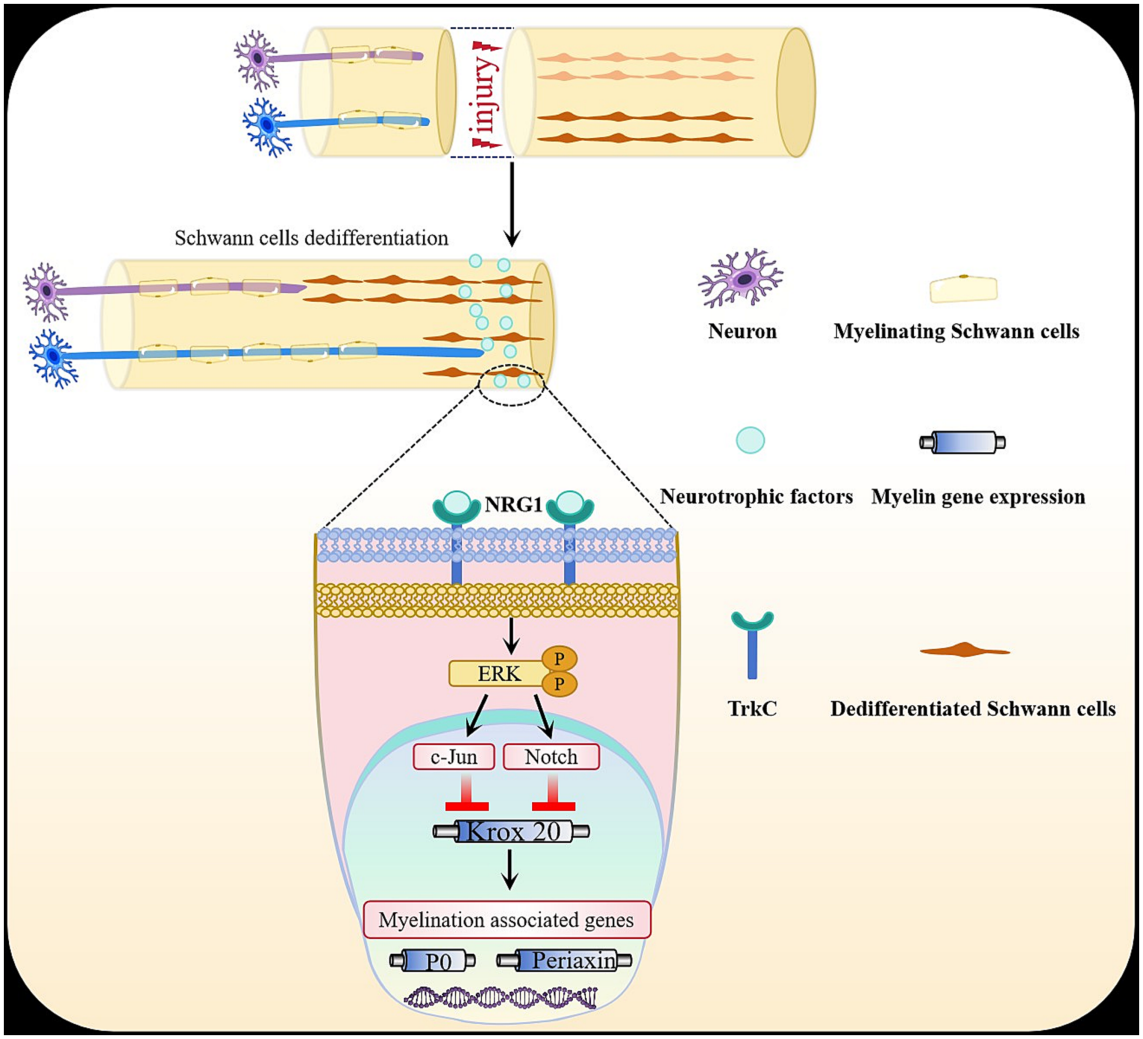
Crosstalk between ERK signaling pathway and other myelination regulators. The ERK signaling pathway may interact with other pathways that control myelination. C-Jun has been recognized as a negative regulator of myelination. Notch signaling, via its intracellular domain (NICD), also functions as a negative regulator of myelination. Strong upregulation of c-Jun and the Notch ligand jagged-1 occurs in Schwann cells after Raf fstimulation, indicating that c-Jun and Notch pathway are downstream of the ERK signaling pathway. C-Jun and Notch pathway inhibit Schwann cell differentiation and myelination by interfering with Krox20 activity, thus repressing peripheral myelination transcription such as periaxin and P0.

Recently, the possibility that the ERK signaling pathway may interact with other pathways that control myelination has been elevated in investigations on Schwann cell development, myelination, and peripheral nerve repair (Figure 4). C-Jun, an important constituent of the AP-1 transcription factor complex, has been recognized as a negative regulator of myelination (Jessen and Mirsky, 2008; Heinen et al., 2013). A previous study in co-cultures revealed that induction of myelin genes is suppressed in Jun-enforced Schwann cells, whereas the expression of myelin genes is elevated in c-Jun null Schwann cells (Parkinson et al., 2008). U0126, a highly selective ERK signaling inhibitor, was found to decrease c-Jun upregulation in the sciatic nerve, suggesting an interaction between the ERK1/2 and JNK pathways during SC transcription (Lindwall Blom et al., 2014). In differentiated PC12 cells after injury, both ERK1/2 and JNK suppression could suppress c-Jun expression and phosphorylation, implying that c-Jun can be controlled by the Ras/ERK1/2 signaling pathway (Waetzig and Herdegen, 2005). Furthermore, a study in DRG neuron/Schwann cell co-cultures found that by inhibiting the ERK pathway, both demyelination and c-jun expression induced by NRG1 were suppressed (Syed et al., 2010). In Schwann cells, NT-3 was found to upregulate c-Jun, mainly through the ERK pathway (Xu et al., 2023). Notch, a transmembrane receptor protein, also functions as a negative regulator of myelination (Jessen and Mirsky, 2008; Heinen et al., 2013). *In vitro* studies showed that upregulating the myelin proteins periaxin and P0 through enforced Krox20 expression was blocked by simultaneous infection with Ad-Notch intracellular domain (NICD). Another *in vivo* experiment revealed that in mice, elevating Schwann cell NICD expression temporarily around birth causes a delay in myelination (Woodhoo et al., 2009). Strong upregulation of c-Jun and the Notch ligand jagged-1 occurs in Schwann cells after Raf stimulation, indicating that the Notch pathway and c-Jun are downstream of the ERK signaling pathway (Napoli et al., 2012) (Figure 4). Concurrently, increased ERK1/2 phosphorylation in sciatic nerves was shown to be associated with a substantial increase in Notch1 receptor gene expression (Nasser et al., 2022). In summary, strong upregulation of c-Jun and the Notch ligand jagged-1 occurs in Schwann cells after Raf stimulation. C-Jun and Notch pathway inhibit Schwann cell differentiation and myelination by interfering with Krox20 activity, thus repressing peripheral myelination transcription such as periaxin and P0 (Figure 4). Furthermore, a study indicated that eIF2α phosphorylation exerts a protective function in CMT1B Schwann cells by limiting ERK/c-Jun hyperactivation (Scapin et al., 2020). However, the precise mechanism still needs to be further studied.

## ERK signaling pathway and peripheral neuropathies

ERK signaling pathway has a function in the progression of both hereditary and acquired peripheral neuropathies, including inherited disorders that cause demyelination or Schwann cell plasticity in damaged or diseased nerves. Charcot–Marie Tooth neuropathy is an inherited demyelinating neuropathy (Berger et al., 2006). In the mouse model of Charcot–Marie Tooth neuropathy, the ERK signaling pathway was activated within the nuclei of certain myelinating Schwann cells, which is directly related to elevated levels of the macrophage-attracting cytokine MCP-1 in the nerves, identifying ERK signaling pathway as a crucial intracellular pathway linking the Schwann cell mutations to the activation of macrophages that are pathogenetically significant in the peripheral nerves (Fischer et al., 2008). Neurofibromatosis type 1 (NF1) is a prevalent genetic condition of the nervous system, inherited in an autosomal dominant manner, impacting one in every 3,500 individuals globally (Rubin and Gutmann, 2005). The loss of neurofibromin was found to induce Ras hyperactivation and its downstream effectors (Cichowski and Jacks, 2001). In NF1−/− and malignant peripheral nerve sheath tumor (MPNST)-derived Schwann cells, the expressions of active Ras were increased and proven to be vital for sustaining the transformed phenotype in these cells (Declue et al., 1992; Basu et al., 1992; Kim et al., 1995). In primary co-culture systems, NF1 loss was found to hinder Schwann cells from connecting with axons and cause already connected Schwann cells to detach from axons by upregulating the Ras/Raf/ERK signaling pathway. Moreover, Normal axonal interactions were restored by treating NF1^−/-^Schwann cells with the MEK inhibitor U0126 after losing axonal interactions (Parrinello et al., 2008). In a three-dimensional *in vitro* culture, the findings demonstrated that hyperactivation of focal adhesion kinase (FAK) leads to the aberrant activation of ERK and AKT downstream of Ras, allowing human NF1-deficient and mouse Nf1−/− cells to proliferate inside a three-dimensional matrix. Furthermore, the study demonstrated that combining both the MEK inhibitor Selumetinib and the FAK inhibitor defactinib entirely inhibits their transformational potential, offering novel approaches for treating plexiform neurofibroma (Errico et al., 2021). These findings not only clarify a molecular mechanism for tumorigenesis but also present a novel strategy for developing NF1 therapies. Moreover, the ERK signaling pathway is associated with immune-mediated peripheral nerve injury caused by leprosy bacilli. In research on the response of human primary Schwann cells to prolonged *Mycobacterium leprae* infection, it was found that intracellular *M. leprae* directly stimulated ERK1/2 via a PKC-dependent and MEK-independent signaling pathway, thereby inducing continuous proliferation (Fang et al., 2015). Diabetic peripheral neuropathy is a kind of acquired demyelinating disease. The DRG and sciatic nerves showed elevated ERK phosphorylation in diabetic animal models (Stavniichuk et al., 2013). *In vitro*, high glucose levels also triggered the activation of ERK in cultured SCs in a dose-dependent way (Zhu et al., 2012). Furthermore, in primary-culture Schwann cells subjected to elevated glucose levels and in Schwann cell-dorsal root ganglion (DRG) neuron co-cultures under similar conditions, the inhibition of the ERK signaling pathway prompted Schwann cell differentiation, increased CNTF release, augmented both protein and mRNA expressions of myelin, and improved the quantity and length of myelin segments (Liu et al., 2020). These findings suggest that the ERK signaling pathway may play a role in the development of diabetic peripheral neuropathy. In addition, the contribution of the ERK signaling pathway activation to neuropathic pain has been observed in mice after partial sciatic nerve ligation, implying that p-ERK and mediators associated with ERK could be targeted for treating neuropathic pain (Kiguchi et al., 2009). A study conducted in a TRPV4-mediated trigeminal neuralgia rat model observed that the ERK antagonist treatment could reverse the mechanical hyperalgesia threshold, nerve fiber abnormalities, myelin degradation, and Schwann cell proliferation (Liu et al., 2021). Furthermore, a recent study reported that miR-30b-5p contained within skin-derived precursor Schwann cells (SKP-SC-EVs) promoted the regeneration of injured sciatic nerves and supported axon growth and myelination in dogs by activating the phosphorylation of ERK, suggesting that SKP-SC-EVs-incorporating TENGs represent an innovative bioactive material that could be used for peripheral nerve repair in clinical practice (Yu et al., 2024).

## Conclusions and future perspectives

Myelination is a complex process and is determined by a balance between positive and negative factors. Recent studies have demonstrated that the ERK signaling pathway is essential for developmental PNS myelination and post-injury remyelination, encompassing the regulation of Schwann cell differentiation, myelination, dedifferentiation, proliferation, remyelination, inflammatory responses, neurotrophic factors, and axon regeneration. Nonetheless, the findings must be contextualized, as varying degrees of ERK activity delineate the state of Schwann cell differentiation. In addition, the ERK signaling pathway may potentially interact with other pathways that control Schwann cell myelination, including c-Jun and Notch. Understanding how these signaling pathways control alterations in the transcriptional network that regulates Schwann cell activity will be tough. The dysregulation of the ERK signaling pathway has been demonstrated to contribute to the development of peripheral neuropathies. Nonetheless, the role of the ERK signaling pathway in human neuropathic disorders is not yet fully understood. Additional investigation in suitable animal models and humans is essential to assess the function of the ERK signaling pathway in the pathogenesis of hereditary and acquired peripheral nerve disorders and to determine if the restoration of ERK signaling pathway regulation could facilitate effective remyelination and regeneration. Enhanced comprehension of the molecular mechanisms governing the unique functions of the ERK signaling pathway in modulating Schwann cell myelination would aid in the formulation of novel therapeutic approaches for severe disorders associated with dysregulated myelination.
